# Giving time a chance in the midsession reversal task

**DOI:** 10.3758/s13420-023-00606-z

**Published:** 2023-11-20

**Authors:** Catarina Soares, Carlos Pinto, Armando Machado

**Affiliations:** 1 School of Psychology, University of Minho, Gualtar Campus, 4710-057 Braga, Portugal; 2 William James Center for Research, University of Aveiro, 3810-193 Aveiro, Portugal

**Keywords:** Timing, Reinforcement contingencies, Midsession reversal task, Pigeon

## Abstract

The midsession reversal task involves a simultaneous discrimination between stimuli S1 and S2. Choice of S1 but not S2 is reinforced during the first 40 trials, and choice of S2 but not S1 is reinforced during the last 40 trials. Trials are separated by a constant intertrial interval (ITI). Pigeons learn the task seemingly by timing the moment of the reversal trial. Hence, most of their errors occur around trial 40 (S2 choices before trial 41 and S1 choices after trial 40). It has been found that when the ITI is doubled on a test session, the reversal trial is halved, a result consistent with timing. However, inconsistent with timing, halving the ITI on a test session did not double the reversal trial. The asymmetry of ITI effects could be due to the intrusion of novel cues during testing, cues that preempt the timing cue. To test this hypothesis, we ran two types of tests after the regular training in the midsession reversal task, one with S1 and S2 choices always reinforced, and another with S1 always reinforced but S2 reinforced only after 20 trials when the ITI doubled or 40 trials when the ITI halved. For most pigeons, performance was consistent with timing both when the ITI doubled and when it was halved, but some pigeons appeared to follow strategies based on counting or on reinforcement contingencies.

To study animal intelligence across taxa, few experimental protocols have been more fruitful than the serial reversal learning task (Bitterman, 1965, 1975; Bond et al., 2007; Mackintosh et al., 1968; Ploog & Williams, 2010; Shettleworth, 2010). One of its versions, the midsession reversal task (MSR), has received considerable attention lately because it has revealed interesting species differences and surprising behavioral strategies. In a MSR task (e.g., Cook & Rosen, 2010; Rayburn-Reeves et al., 2011), the animal learns a simultaneous discrimination involving two stimuli, S1 and S2. For the first 40 trials, only S1 choices are rewarded (i.e., S1^+^/S2^−^). For the next 40 trials only S2 choices are rewarded (i.e., S1^−^/S2^+^). That is, the reinforcement contingencies reverse at midsession, on trial 41. To learn this task, animals can use different strategies. A highly efficient strategy is win-stay/lose-shift: Choose S1 while it is reinforced and switch to S2 following the first unreinforced S1 choice on trial 41. Another strategy is to learn first the number of the reversal trial, and then compare a memory of that number, n*, with the current trial number, n, choosing S1 if n is less than n*, and S2 otherwise. A third strategy, similar to the number-based strategy, exploits the temporal regularities of the task: Because trials are separated by short and constant intertrial intervals (ITI) and choice latencies remain typically below 1 s, the trials proceed regularly and the reversal trial tends to occur at approximately the same moment into each session. The timing strategy is to learn first the moment of the reversal trial, and then compare the memory of that moment, t*, with the time elapsed since the beginning of the session, t, choosing S1 while t is less than t*, and S2 once t becomes greater than t*.

The three strategies engender different psychometric functions relating preference for S1 to trial number. Let p(n) stand for the probability of choosing S1 on trial n. If an animal follows the win-stay/lose-shift strategy, we expect p(n) to remain close to 1 until the reversal trial and close to 0 thereafter; in other words, p(n) should resemble a step function with the step after trial 41. The abrupt change in preference would express the distinctiveness of the first unreinforced choice. In contrast, if the animal follows a counting or timing strategy, we expect p(n) to decrease from 1 to 0 more gradually than the step function, for the discrimination of trial number or time follows Weber’s law. In both cases, we expect errors around trial 41, anticipation errors (choices of S2 shortly before trial 41) and perseveration errors (choices of S1 shortly after trial 40). However, the point of subjective equality (PSE)—the point where subjects are indifferent between the two stimuli (p(n) ≈ 0.5) should remain close to trial 40.

The MSR task has been studied across several species such as pigeons (e.g., Rayburn-Reeves et al., 2011), rats (e.g., Rayburn-Reeves, Stagner, et al., 2013), humans (e.g., Rayburn-Reeves et al., 2011), dogs (Laude et al., 2016), rhesus macaques (e.g., Rayburn-Reeves et al., 2017), starlings (Machado et al., 2023), and, with modified versions, chickadees (McMillan, Hahn, et al., 2017) and kea (Laschober et al., 2021). These studies revealed interspecies differences in the modal behavioral strategies. For instance, whereas humans (Rayburn-Reeves et al., 2011) and rhesus macaques (Rayburn-Reeves et al., 2017) tend to follow the win-stay/lose-shift strategy, rats and pigeons seem to adopt different strategies according to experimental details. Rats tend to follow a strategy closer to win-stay/lose-shift when presented with a spatial discrimination between two levers in a typical operant chamber (e.g., Rayburn-Reeves, Stagner, et al., 2013; Rayburn-Reeves et al., 2018; Santos & Sanabria, 2020; Smith et al., 2016). But when the discrimination is between the two arms of a T-maze and the rats are placed in the start box at the beginning of each trial, they seem to adopt either a counting or timing strategy (McMillan et al., 2014).

Pigeons, the species most studied in the MSR task, tend to adopt either a counting or a timing strategy when the discrimination is visual (e.g., S1 and S2 are red and green keylights; see Laude et al., 2014; McMillan & Roberts, 2012; Rayburn-Reeves et al., 2011; Rayburn-Reeves, Laude, et al., 2013), or when the discrimination is spatial (e.g., S1 and S2 are left and right keys) and the ITI is relatively long (e.g., 5 or 10 s; see Laude et al., 2014; Rayburn-Reeves, Laude, et al., 2013). But they tend to follow a win-stay/lose-shift strategy when either the ITI is very short (e.g., 1.5-s, Rayburn-Reeves, Laude, et al., 2013; Laude et al., 2014), or the discrimination is visual-spatial (e.g., S1 and S2 are left-red and right-green keys; McMillan & Roberts, 2012; McMillan et al., 2014). For a review, see McMillan, Spetch et al. (2017), Rayburn-Reeves and Cook (2016) or Zentall (2020).

Several studies show that, of the two strategies, counting and timing, pigeons are more likely to adopt the timing strategy. Cook and Rosen (2010) trained pigeons in a MSR task in which during the first 48 trials, pigeons were required to perform a matching-to-sample task with two color stimuli and, in the following 48 trials, the task was changed to oddity-from-sample. During test sessions, a temporal gap was introduced before the reversal (between trials 20 and 21) and pigeons changed their behavior from matching to oddity according to the duration of the gap—the longer the temporal gap, the earlier the trial the change happened. In a following phase, the authors trained pigeons with time-based reversals—for instance, 40-min sessions with reversal at 20-min—and, when stable, performance was well described by a psychometric function similar to that observed when the reversal was based on trial number, with the PSE at approximately the temporal midsession. Then, pigeons were tested with nondifferential reinforcement (i.e., all responses were reinforced) and switching still occurred close to the previously-learned reversal time.

More direct evidence of timing rather than counting in the MSR task comes from studies that changed the ITI duration from training to testing and therefore dissociated the reversal *moment* from the reversal *trial*. Thus, McMillan and Roberts (2012, Phase 2; see also Smith et al., 2017) trained a group of pigeons in a MSR task with a color discrimination and a 6-s ITI. Then, for the next 20 sessions, the ITI in each fourth session doubled to 12 s. Finally, during the following 20 sessions, the ITI in each fourth session was halved to 3 s. If the pigeons were following a win-stay/lose-shift or a number-based strategy, the ITI change should not affect the trial on which the pigeons switched preference from S1 to S2. However, if the pigeons followed a time-based strategy, the ITI change should affect the reversal trial. Specifically, with an ITI twice as long, the moment of reversal occurs around trial 20, twice as early, and, conversely, with the ITI twice as short, the moment of reversal occurs around trial 80, twice as late. Results showed that when the ITI doubled, pigeons did switch preference around trial 20, but when the ITI was halved, pigeons switched preference around trial 50, later than expected if they were following a win-stay/lose-shift, or a counting strategy, but significantly earlier than predicted if they were timing. Soares et al. (2020) reproduced these results with a between-subjects design. The group that experienced during testing an ITI twice as long switched preference twice as early, but the group that experienced an ITI twice as short did not switch preference twice as late.

This asymmetry of the ITI effects may be due to interference between cues (e.g., Soares et al., 2020). In test sessions with an ITI twice as long, the pigeon experiences the same events as during training (i.e., choosing S1 and receiving food) until, around trial 20, the timing cue directs it to switch preference. In other words, during the first 20-odd trials, no cue intrudes and preempts the timing cue. Although the pigeon will not receive food for choosing S2 from trials 21 to 40, the continuous extinction of S2 choices (the novel cue) will have followed rather than preceded the (presumably time-based) switching of preference. In short, when the ITI doubles, extinction cues do not interfere with the initial expression of timing-based behavior. The case is otherwise when the ITI is halved in the test session. The timing cue directs the pigeon to switch preference around trial 80. However, after the first 40 trials, choosing S1 will result in extinction. Repeated extinction of S1 choices past trial 40 introduces a novel cue that preempts the timing cue. Hence, when the ITI is halved, time is not given a chance to exert its influence over choice. Additionally, even though in both tests there is a period during which time-into-the-session and response-outcome events cue different choices (i.e., time-based choices are not reinforced), this “conflicting” period is shorter for the double-ITI test (approximately from trial 20 to 40) than for the half-ITI test (approximately from trial 40 to 80); this difference may also contribute to the asymmetry between tests.

To test the foregoing account, the test sessions should be such that the temporal control is not preempted by extraneous, intrusive cues. Thus, in the present study we ran two versions of the MSR task in which we attempted to reduce the intrusion of other cues on the expression of timing. In the first version, we reinforced all choices during the test session, as Cook and Rosen (2010) did in their time-based version of the MSR task. Specifically, we trained two groups of pigeons, one with a 5-s ITI and another with a 10-s ITI. Then, in the test session, we doubled the ITI for the first group and halved it for the second. We also extended the test sessions to 120 trials to give the timing cue a chance to exert its effects, if any, around trial 80, the predicted trial when the ITI was halved. If cue interference is indeed reduced by reinforcing all choices during the test session, the group with an ITI twice as long should produce a psychometric function with a PSE around trial 20 (McMillan & Roberts, 2012; Smith et al., 2017; Soares et al., 2020), whereas the group with an ITI twice as short should produce a psychometric function with the PSE around trial 80.

Reinforcing all choices during testing may reduce the effect of extinction cues, but only at the cost of introducing reinforcement effects that counter the timing strategy. One such effect could be the reinforcement of S2 choices during the first trials—that is, when S2 choices are unlikely to be time based. Although the timing cues direct the pigeon to choose S1, reinforcement contingencies could strengthen choices of S2. We attempted to reduce these effects with the second version of the MSR task. During the test session S1 responses were always reinforced, as in the first version, but with the doubled ITI S2 responses were reinforced only after trial 20, and with the halved ITI S2 responses were reinforced only after trial 40. To the extent that we reduce both sources of cue interference, extinction of S1 choices and reinforcement of highly premature S2 choices, the psychometric functions will shift in the expected direction and magnitude, halving the PSE when the ITI is doubled, and doubling the PSE when the ITI is halved.

## Method

### Subjects

Twelve pigeons (*Columba livia*) participated in the experiment, divided into two groups of six. The sample size (six per group) was determined by an a priori power analysis (G*Power 3.1) using a *t* test for related samples to compare the PSEs from training and testing sessions, with a significance level of .05 (two-tailed), power of .80, and an estimated effect size, based on a previous study (Soares et al., 2020), of at least 1.5. All pigeons had experience with temporal and numerical discriminations but no experience with the MSR task.

The pigeons were maintained at approximately 85% of their free-feeding weight. They were housed individually in a temperature-controlled colony room (between 21° and 22° C) on a 13:11 h light/dark cycle, with lights on at 8 a.m. Grit and water were always available in their home cage. The pigeons were cared for in accordance with the animal care guidelines of the Portuguese Directorate–General for Food and Veterinary, and of the University of Minho.

### Apparatus

The experiment was conducted in four operant chambers for pigeons: three LVE (Lehigh Valley Electronics) chambers and one homemade chamber. The LVE chambers measured 34 × 35 × 31 cm (height × length × width) and were equipped with an exhaust fan that circulated air through the chamber and masked outside noises. All walls were made of aluminum and a wire mesh served as the floor. On the front panel, there were three horizontally aligned circular keys, each 2.5-cm in diameter, and placed 22.5 cm above the floor and 8.5 cm apart, center to center. The keys were equipped with a 12-stimulus IEE (Industrial Electronics Engineers) in-line projector with each stimulus illuminated with a 28-V, 0.1-A lamp. Mixed grain was delivered by a LVE food hopper accessible through a 6-cm wide × 5-cm high opening centered horizontally in the front panel, below the keys and 8.5-cm above the floor. When a reinforcer was delivered, the hopper was raised and a 28-V, 0.04-A light illuminated the grain.

The homemade chamber (31 × 33 × 33 cm) was enclosed in a PVC sound-attenuating cubicle (Med Associates, ENV-018V) equipped with an exhaust fan. All walls, with the exception of the front wall, were made of acrylic and a wire mesh served as the floor. On the front panel, there were three horizontally-aligned circular keys, 2.5 cm in diameter, 9 cm apart, center to center, and 21 cm above the floor. The keys were equipped with a 12-stimulus IEE in-line projector with each stimulus illuminated with a 28-V, 0.1-A lamp. An LVE food hopper delivered mixed grain through a 6-cm wide × 4.5-cm high opening, centered horizontally in the front panel, 6.5 cm above the floor. When the hopper was raised, the grain was illuminated with a 28-V, 0.04-A light. Experimental events were controlled and recorded by a personal computer using ABET II^®^ software (Lafayette Instruments).

### Procedure

The pigeons were divided randomly in two groups of equal size. One group learned the MSR task with a 5-s ITI and was then tested with a 10-s ITI, and the other group learned the MSR task with a 10-s ITI and then was tested with a 5-s ITI. During this test session, S1 and S2 choices were always reinforced. We refer to these train + test conditions as Condition 5-10 (I) and Condition 10-5 (I), respectively, with the first and second numbers standing for the training and testing ITI durations. Then, each group repeated its initial training and testing conditions except that, as explained below, during the test sessions not all S2 choices were reinforced. We refer to these train + test conditions as Condition 5-10 (II) and Condition 10-5 (II). Finally, each group experienced the opposite train + test condition (i.e. pigeons in Condition 5-10 (II) moved to Condition 10-5 (II) and vice versa). We detail each condition next.

#### Condition 5-10 (I)

Sessions occurred 6 days a week at approximately the same time of day for each pigeon. They started with a free reinforcer for 3 seconds. Each session comprised 80 simultaneous color discrimination trials. At the beginning of a trial, the two side keys were illuminated, one with a green hue and the other with a red hue; hue position was counterbalanced across trials so that in each session each hue appeared the same number of times on the left and right keys. From trials 1 to 40 only responses to S1 were reinforced, and from trials 41 to 80 only responses to S2 were reinforced. The assignment of red and green colors to S1 and S2 were counterbalanced across pigeons. The first peck to either key turned both keys off. If the response was correct, the food hopper was raised and illuminated for 2 s after which a 3-s interval, spent in darkness, started. If the response was incorrect, a 5-s interval spent in darkness started. Thus, regardless of whether the trial ended in food or not, the effective ITI was always 5-s long. This phase lasted a minimum of 20 sessions, and it continued until the proportion of correct responses in each half of the session was at least .80 for three consecutive sessions, or until a maximum of 30 sessions was reached.

After training, the pigeons were exposed to a single test session in which the effective ITI doubled to 10 s. Given that on every trial, regardless of the pigeon’s choice, a 2-s reinforcer followed, the interval spent in darkness after food lasted 8 s. The test session comprised 120 trials, so session duration increased from approximately 8 min during training to 22 min.

#### Condition 10-5 (I)

Except for the ITI, this condition was in all aspects similar to Condition 5-10 (I). During training the effective ITI was 10-s long, and during the test session it was 5-s long. A session lasted approximately 15 min during training and 12 min during testing.

#### Condition 5-10 (II)

Training was the same as in Condition 5-10 (I). The test differed in that S1 choices were always reinforced, but S2 choices were reinforced only after trial 20, the trial in which time since the beginning of the session approximately matched the time of the reversal trial during training.

#### Condition 10-5 (II)

Training was the same as in Condition 10-5 (I). The only difference occurred in the test session, S1 choices were always reinforced but S2 choices were reinforced only after trial 40, the trial in which choices of S2 were reinforced during training.

### Data analysis

For each condition, we plotted the psychometric function relating the proportion of S1 choices as a function of five-trial blocks, for the last session of training and for the test session. To calculate the trial-based PSEs, we fitted Gaussian distributions with two free parameters (mean and standard deviation) to the individual psychometric functions using the least-squares method, and then used the mean parameter as the PSE. The PSE is not presented for psychometric function that either did not cross the indifference line or fit the data poorly (*R*^2^ < .50). For Conditions 5-10 (II) and 10-5 (II), we also estimated time-based PSEs by identifying when in the session each trial-based PSE occurred, and computed the ratios between training and test PSEs. For trial- and time-based PSEs and their ratios we constructed 95% Confidence Intervals (CI) for the mean, using the *t* distribution.

Statistical tests were conducted using IBM SPSS Statistics for Windows (Version 28) with Type I error rate set at 0.05. We used paired-samples *t* tests to compare the switching trials (i.e., the trial-based PSE) and switching times (time-based PSE) between training and testing. Given that Conditions 5-10 (II) and 10-5 (II) were counterbalanced among pigeons, to test for order effects we compared performance in each phase via repeated-measures ANOVAs. As measures of effect size, we reported the standardized mean difference for the paired *t* tests (*d*_z_, e.g., Cohen, 1988, p. 48; Lakens, 2013) and the generalized eta square for the ANOVAs (${\upeta}_G^2$, e.g., Bakeman, 2005; Olejnik & Algina, 2003).

## Results

### Condition 5-10 (I)

**Fig. 1:**
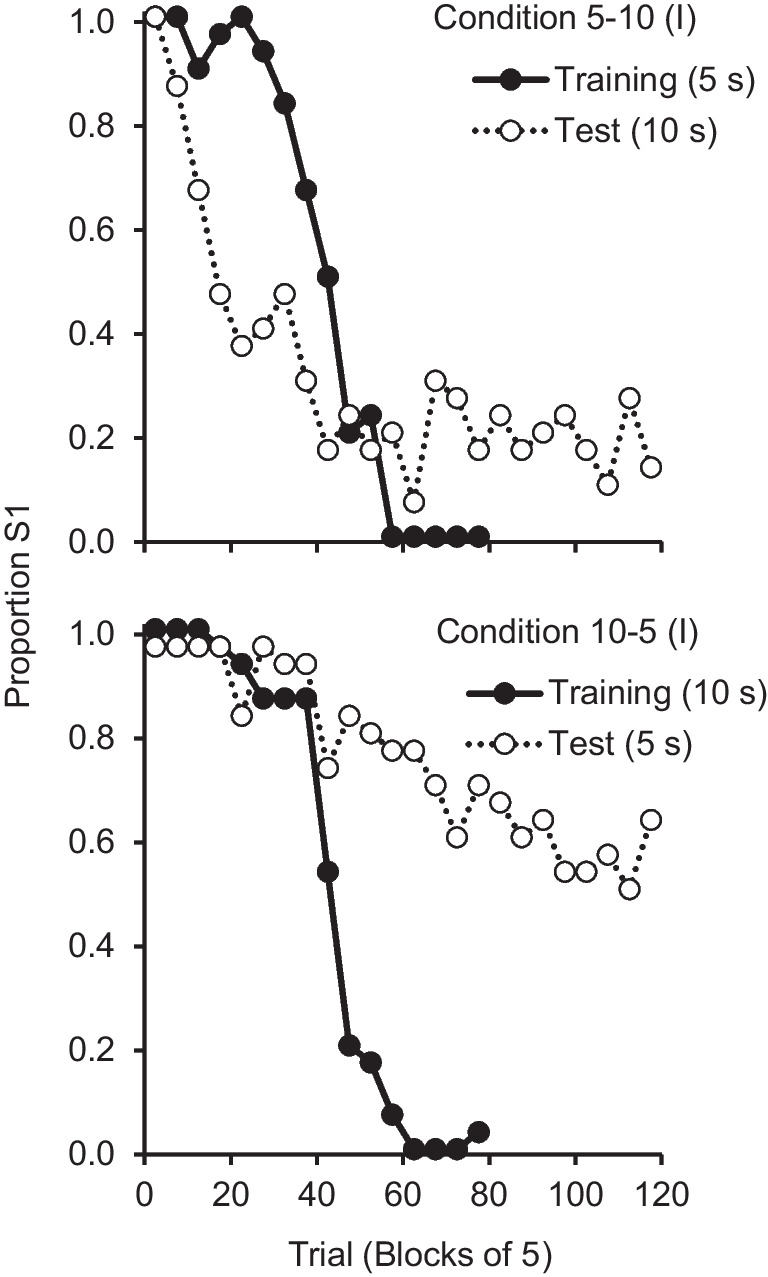
Average psychometric functions for the last session of training and the test session from Conditions 5-10 (I) and 10-5 (I)

All pigeons completed training in 20 or 21 sessions (average = 20.17). The top panel of Fig. 1 shows the averaged proportion of S1 choices as a function of five-trial blocks, in the last session of training (5-s ITI) and in the test session (10-s ITI). In the last session of training (filled circles), the pigeons started the session with a strong preference for S1, switched preference around trial 42, close to the reversal trial, and ended the session with a strong preference for S2. The individual and average psychometric functions had the usual sigmoid shape, a shape well fitted by a Gaussian curve. Table 1 shows the best-fitting PSEs. In the test session (unfilled circles), with an ITI twice as long, preference switched on average by trial 19. However, there was more variability at the individual level (Fig. 3, Appendix). For two pigeons (P123 and P902), the psychometric function after the first few trials oscillated around indifference such that the Gaussian curve could not fit the data well (*R*^2^ < .50). For these pigeons, a PSE could not be estimated. For the remaining four pigeons, the psychometric functions were more regular, and the Gaussian fit them better; a paired-samples *t* test showed that their estimated trial-based PSEs during the test session (*M* = 19.25) differed significantly from their PSEs in the last session of training (*M* = 40.75), *t*(3) = 5.87, *p* = .01, *d*_z_ = 2.93.

**Table 1:** Estimated trial-based PSE in the last session of training and in the test session of Conditions 5-10 (I) and 10-5 (I)

Condition 5-10 (I)				Condition 10-5 (I)			
Pigeon		Training	Test	Pigeon		Training	Test
P123		42	–	P069		47	–
P709		33	22	P192		42	–
P902		47	–	P452		41	105
P974		47	20	P474		40	–
P9131		43	21	P960		43	58
PG39		40	14	PG40		45	–
Avg.		42.00	19.25	Avg.		43.00	–
Std.		5.22	3.59	Std.		2.61	–
95% CI	LL	36.53	13.53	95% CI	LL	40.26	–
	UL	47.47	24.97		UL	45.74	–

*Note.* The last four rows show the average (Avg.), standard deviation (Std.), and lower limit (LL) and upper limit (UL) of the 95% confidence interval (CI) for the average PSE

### Condition 10-5 (I)

The pigeons completed training in 20 to 23 sessions (average = 21.33). The bottom panel of Fig. 1 displays the average results. In the last session of training with a 10-s ITI, the individual and average psychometric functions had the usual sigmoid shape with a trial-based PSE close to the reversal trial (average PSE = 43.00; see Table 1 for individual PSEs). In the test session with an ITI half as long, the average function decreased across trials, but it did not represent well the individual functions (see Fig. 4, Appendix). There was considerable interindividual variability and for most pigeons the PSE could not be estimated (see Table 1)—either the psychometric function never crossed indifference or a Gaussian curve did not fit the data well (*R*^2^ < .50). Two pigeons (P474 and PG40) appeared to become indifferent between the two options—as seen in Condition 5-10 (I)—whereas two others (P069 and P452) chose S1 on most trials, and one pigeon (P192) chose S1 on all trials. Only P960’s psychometric function followed the usual sigmoid pattern, although shifted to the right.

### Condition 5-10 (II)

To assess whether performance differed between the pigeons that experienced Condition 5-10 (II) first and those that experienced it after Condition 10-5 (II), an ANOVA with five-trial bin (16 levels) as within-subject factor and order (two levels) as between-subject factor was run for the last session of training (ITI = 5 s). There was no significant factor interaction, *F*(15, 150) = 0.82, *p* = .658, ${\upeta}_G^2=$ 0.06, nor main effect of order, *F*(1, 10) = 0.02, *p* = .896, ${\upeta}_G^2=$ 0.0003. For the test data (ITI = 10 s), an ANOVA with five-trial bin (24 levels) as within-subject factor and order (two levels) as between-subject factor revealed similar results: no significant factor interaction, *F*(23, 207) = 0.35, *p* = .998, ${\upeta}_G^2=$ 0.034, nor main effect of order, *F*(1, 9) = 3.58, *p* = .091, ${\upeta}_G^2=$ 0.036. Given that the two groups of pigeons did not perform differently, we combined their data.

**Fig. 2:**
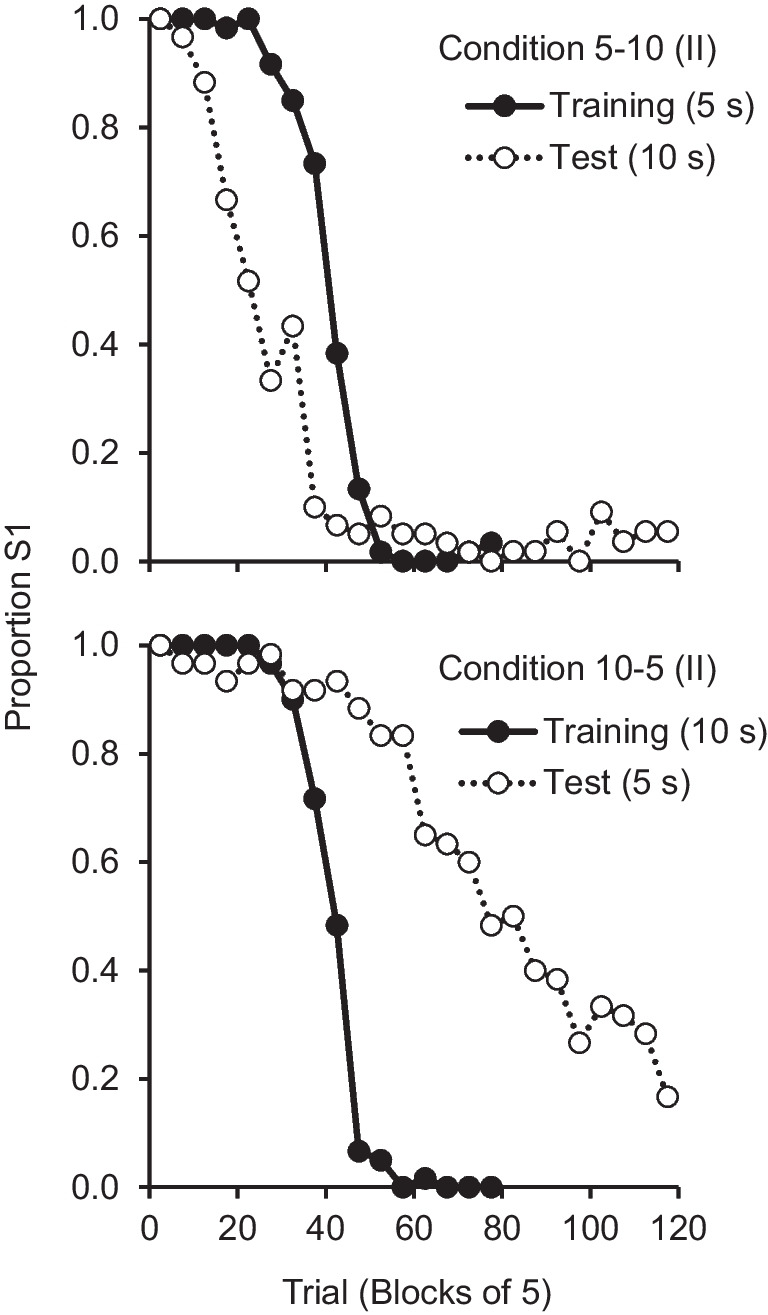
Average psychometric functions for the last session of training and the test session from Conditions 5-10 (II) and 10-5 (II)

**Fig. 3:**
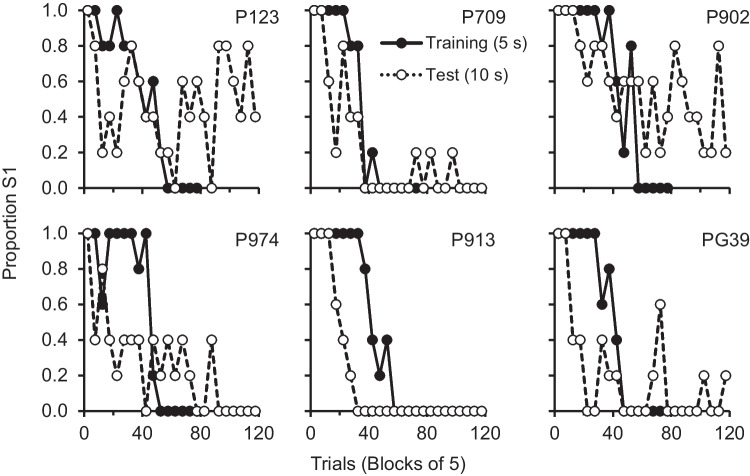
Individual psychometric functions from training and testing in Condition 5-10 (I)

Training in Condition 5-10 (II) ranged from 20 to 30 sessions (average = 21.25). Pigeon P960 completed only 77 trials of the test session, due to a power failure in the experimental room, but it was still possible to identify its reversal moment in the session. By the end of training, the psychometric functions had the typical sigmoid shape, with PSE close to the reversal trial (see Table 2 for the individual parameters). In the test session, contrary to what was observed in Condition 5-10 (I), preference for S1 remained close to zero after trial 40—the procedural changes appeared to reduce the issues observed before. For one pigeon (P123), a Gaussian curve did not fit the data well (*R*^2^ < .50) and the test PSE was not estimated. Figure 2 (top panel) shows the average curves. In the last training session, pigeons switched preference close to the reversal trial—average PSE = 40.33, 95% CI [37.44, 43.23]—approximately 235 s into the session. In the test session, preference reversed shortly after trial 20—average PSE = 25.27; 95% CI [20.96, 29.59]—approximately 271 s into the session. The switching trial differed between training and testing, *t*(5) = 5.54, *p* < .001, *d*_z_ = 1.67, but the switching time did not, *t*(5) = 1.39, *p* = .195, *d*_z_ = 0.42.

**Table 2:** Estimated trial- and time-based PSEs from the last session of training and the test session of Conditions 5-10 (II) and 10-5 (II)

Pigeon		Condition 5-10 (II)						Condition 10-5 (II)					
		Trial			Time (s)			Trial			Time (s)		
		Train	Test	Ratio	Train	Test	Ratio	Train	Test	Ratio	Train	Test	Ratio
P123		40	–	–	223	–	–	37	77	2.08	393	441	1.12
P709		38	24	0.63	209	249	1.19	33	73	2.21	343	412	1.20
P902		48	32	0.67	276	340	1.23	42	43	1.02	448	248	0.55
P974		34	35	1.03	194	377	1.94	42	60	1.43	451	353	0.78
P9131		38	21	0.55	229	225	0.98	45	83	1.84	481	498	1.04
PG39		43	23	0.53	255	271	1.06	41	95	2.32	456	574	1.26
P069		47	16	0.34	274	167	0.61	43	95	2.21	463	581	1.25
P192		42	26	0.62	245	272	1.11	42	–	–	451	–	–
P452		41	15	0.37	250	158	0.63	39	–	–	413	–	–
P474		35	31	0.89	200	333	1.67	45	80	1.78	479	455	0.95
P960		43	30	0.70	257	320	1.24	40	73	1.83	436	464	1.06
PG40		35	25	0.71	203	273	1.34	40	–	–	432	–	–
Avg.		40.3	25.3	0.64	234.6	271.4	1.18	40.8	75.4	1.86	437.2	447.4	1.02
Std.		4.6	6.4	0.20	28.9	69.6	0.39	3.4	16.4	0.42	38.9	104.1	0.23
95% CI	LL	37.4	21.0	0.50	216.2	224.6	0.92	38.5	62.8	1.54	411.3	367.3	0.85
	UL	43.2	29.6	0.78	253.0	318.1	1.45	43.0	88.0	2.18	463.0	527.4	1.20

*Note.* Ratio is the test/train ratio of the estimated PSEs. The last four rows show the average (Avg.), the standard deviation (Std.), and the lower limit (LL) and upper limit (UL) of the 95% confidence interval (CI) of the mean

To further check whether the pigeons’ performance was consistent with timing, we computed the ratio of the time-based and trial-based PSEs (see Table 2). Timing predicts a time-based ratio close to 1 and a trial-based ratio close to 0.5. The obtained time-based ratios were consistent with timing: average = 1.18, and 95% CI [0.92, 1.45]. The obtained trial-based ratios tended to be slightly greater than predicted: average = 0.64, 95% CI [0.50, 0.78]. Interestingly, two pigeons, P974 and P474, did not seem to follow a timing strategy: the switching trial was approximately the same during training and testing (P974: 34 and 35; P474: 35 and 31), whereas the switching time increased significantly from training to testing (P974: from 194 s to 377 s; P474: from 200 s to 333 s), suggesting that their behavior was controlled by trial number (see Fig. 5, Appendix).

### Condition 10-5 (II)

An ANOVA with five-trial bin (16 levels) as within-subject factor and order (two levels) as between-subject factor was run for the training (ITI = 10 s) data, to assess whether performance differed between the pigeons that experienced Condition 10-5 (II) first and those that experienced it last. There was no significant factor interaction, *F*(15, 150) = 0.529, *p* = .922, ${\upeta}_G^2=$ 0.045, nor main effect of order, *F*(1, 10) = 1.224, *p* = .294, ${\upeta}_G^2=$ 0.013, so we combined the training data from all 12 pigeons.

Training took between 20 and 24 sessions (average = 20.83). Figure 2 (bottom panel, filled data points) shows that by the end of training the average psychometric curve followed a sigmoid shape, with PSE close to the reversal trial (average PSE = 40.75, 95% CI [38.51, 42.99]; see Table 2 for individual PSEs). The average switching trial was similar to Condition 5-10 (II), (*t*(11) = 0.26, *p* = .800, *d*_z_ = 0.08). The switching time occurred on average 437 s into the session, almost twice as late than in Condition 5-10 (II) (235 s).

In the test session (ITI = 5 s), for nine of the 12 pigeons the psychometric function decreased across trials and had well-defined PSEs. For the remaining three pigeons, the PSE could not be estimated because the function either remained close to 1 (P192 and P452) or oscillated around indifference (PG40) throughout the session (Fig. 6, Appendix). These pigeons had already shown similar response patterns in Condition 10-5 (I). Given the impossibility of estimating their PSEs, the test data for these three pigeons were not included in the analysis. For the remaining nine pigeons, an ANOVA with five-trial bin (24 levels) as within-subject factor and order in which the conditions were run (two levels) as between-subject factor, found no significant interaction, *F*(23, 161) = 0.69, *p* = .850, ${\upeta}_G^2=$ 0.063, nor main effect of order, *F*(1, 7) = 0.72, *p* = .423, ${\upeta}_G^2=$ 0.032. Thus, the data from these nine pigeons were combined.

In the test session (Fig. 2, bottom panel, empty data points), when preference reversed, it did so close to trial 80 (average PSE = 75.44; 95% CI [62.85, 88.04]), approximately 447 s into the session. The switching trial differed between training and testing, *t*(8) = 6.31, *p* < .001, *d*_z_ = 2.10, but the switching time did not, *t*(8) = 0.25, *p* = .813, *d*_z_ = 0.08.

Regarding the PSE ratios, timing predicts a time-based ratio close to 1 and a trial-based ratio close to 2.0. Both obtained values were consistent with timing (for the time-based ratios, average = 1.02 and 95% CI [0.85, 1.20]; for the trial-based ratios, average = 1.86 and 95% CI [1.54, 2.18]). Interestingly, as in Condition 5-10 (II), the psychometric functions for one pigeon (P902) overlapped (PSE = 42 in the last session of training and 43 in the test session), with the switching times differing markedly (448 s vs 248 s), which suggests control by trial number.

## Discussion

Previous studies that manipulated the intertrial interval (ITI) in the midsession reversal (MSR) task (McMillan & Roberts, 2012; Smith et al., 2017; Soares et al., 2020) found that when the ITI is doubled, pigeons change their preference around trial 20—when time since the beginning of the session matches the previously-learned time to switch—and thus is consistent with animals learning the MSR task by timing the moment the contingencies change. However, when the ITI is halved, pigeons change their preference around trial 50—earlier than the previously-learned time to switch. This last result could be revealing that nontemporal cues are also at play, namely extinction cues experienced by choosing S1 after trial 40.

**Fig. 4:**
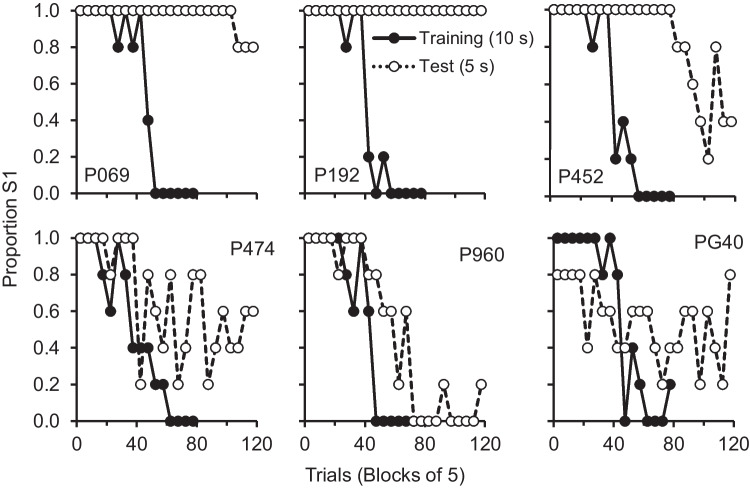
Individual psychometric functions from training and testing in Condition 10-5 (I)

**Fig. 5:**
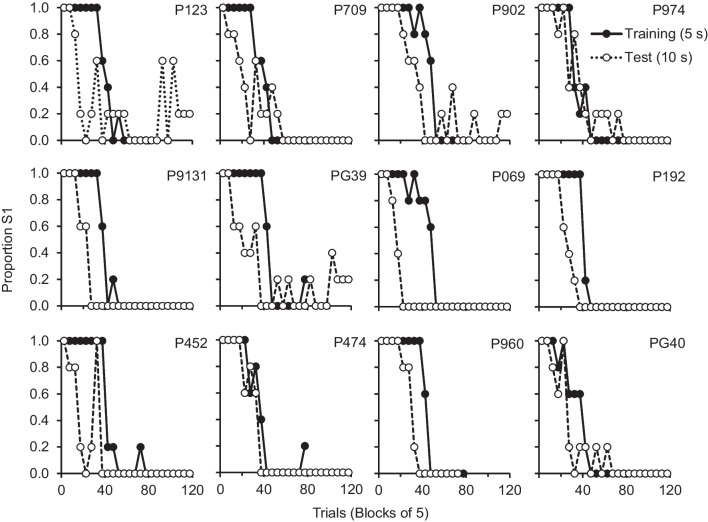
Individual psychometric functions from training and testing in Condition 5-10 (II)*.* ^a^ P960 only completed 77 trials on the test session due to a power cut in the experimental room

**Fig. 6:**
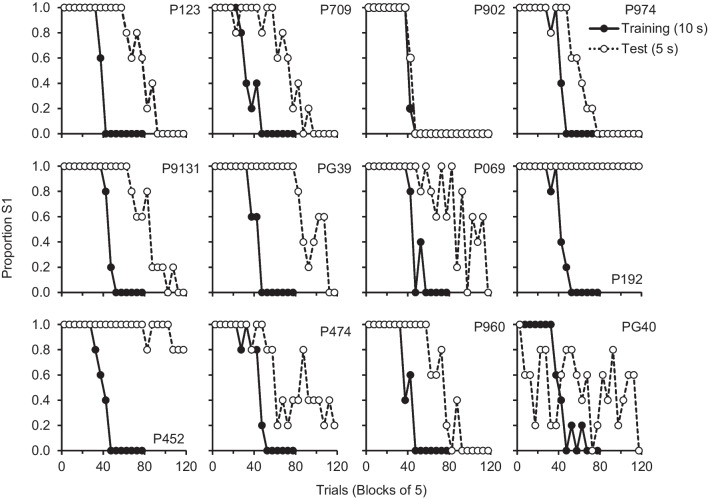
Individual psychometric functions from training and testing in Condition 10-5 (II)

The present study aimed to test this cue interference hypothesis by eliminating the extinction cue. A group of pigeons was trained with a 5-s ITI and tested with a 10-s ITI (Condition 5-10 (I)) whereas another group was trained with a 10-s ITI and tested with a 5-s ITI (Condition 10-5 (I)), with all responses reinforced in the test session. In the test session of Condition 5-10 (I), pigeons switched on average on trial 19 (as expected if the animals were timing) and this result was consistent with previous studies (McMillan & Roberts, 2012; Smith et al., 2017; Soares et al., 2020). However, for two pigeons (P123 and P902) we were unable to estimate the PSE. In the last training session, both pigeons showed anticipatory errors early in the session. In the test session, because all responses were reinforced, these anticipatory errors yielded food and the pigeons alternated between S1 and S2. In the test session of Condition 10-5 (I), when the ITI was halved, with the exceptions of P452 and P960, that switched preference on trial 105 and 58 respectively, we were not able to estimate the PSEs. Two pigeons chose S1 for the entire (or almost entire) session suggesting that they were relying on the absence of reinforcement on S1 to switch to S2—because S1 was always reinforced, the animals never switched. Similar to Condition 5-10 (I), two other pigeons (P474 and PG40) started to choose S2 early in the session and remained indifferent between S1 and S2 for most of the session (see Appendix, Fig. 4). Thus, reinforcement following all responses promoted a tendency to choose randomly and to switch early—in case of anticipatory errors, pigeons experienced reinforcement in S2. These results demonstrate how cue competition can affect performance in this task.

To reduce these potential effects, we modified the reinforcement rules in the test session to prevent reinforcement of early switches to S2 that could erroneously signal that the reversal had already occurred. The modified tests reduced early switching as well as indifference between the two options, such that preference for S1 tended to decrease as the session progressed, and reach values close to or equal to zero until the end of the session. The psychometric functions were consistent with a timing strategy, as pigeons switched preference on the average slightly after the predicted trial of 20 (trial 25) in Condition 5-10 (II) and slightly before the predicted trial of 80 (trial 75) in Condition 10-5 (II). That is, varying the ITI shifted the psychometric functions not only in the direction but also by the degree predicted by timing. When the ITI increased, the PSE decreased by 62% (predicted value of 50%), and when the ITI decreased, the PSE increased by 89% (predicted value of 100%). This is the first time the two ITI tests had effects of similar magnitude. Our findings also provide the strongest evidence to date that pigeons are timing when to switch from S1 to S2.

In the MSR task, there are (at least) three possible sources of behavioral control: The local response-outcome events and the global trial number and elapsed time. The between-subjects variability that we and others observed strongly supports the notion that not all pigeons’ behavior is under control of the same source (see McMillan, Spetch et al., 2017). In the present study, even though our overall results are consistent with a timing strategy, not all birds appeared to follow it. There were pigeons that never reversed preference, choosing S1 exclusively (P192, Conditions 10-5 (I) and 10-5 (II)) or almost exclusively (P069, Condition 10-5 (I); P452, Condition 10-5 (II)), suggesting control by local response-outcome events, given that S1 choices were always reinforced during testing. There were also three birds (P974 and P474 in Condition 5-10 (II); P902, Condition 10-5 (II)) that produced orderly psychometric functions, with anticipatory and perseverative errors (both suggesting global cues), yet with training and test functions overlapping. That is, in the test session these pigeons reversed preference around the same trial as in training, so the reversal moment changed, suggesting that their behavior was under control of trial number. This is particularly interesting since previous studies with this version of the MSR task did not report any instance of control by trial number and because observations of counting by pigeons are typically observed within trials (and not across trials) and with relatively small numerosities. Furthermore, we also observed within-subject changes in strategy across conditions. For instance, the psychometric functions of P192 suggested control by local contingencies in Condition 10-5 (I and II) but by timing in Condition 5-10 (II); the psychometric functions of P452 suggested control by timing in Conditions 10-5 (I) and 5-10 (II) but by local contingencies in Condition 5-10 (II).

To study the interaction of different sources of behavioral control in the MSR task, some studies manipulated the reliability of each of those sources (e.g., Rayburn-Reeves et al., 2011; Santos et al., 2019, 2021; Santos & Sanabria, 2020; Smith et al., 2016; Zentall et al., 2019). For instance, Santos et al. (2019) found that, compared to when reinforcement probability was the same in the whole session, when the probability of reinforcement was lower in the first half (0.20) than in the second half (1.00), there were more anticipatory errors, that is, earlier choices of S2. Thus, pigeons’ behavior was consistent with timing, biased by reinforcement contingencies. However, when the probability of reinforcement was higher in the first half (1.00) than in the second (0.20), the psychometric function did not shift in the opposite direction, which would reduce anticipatory and increase perseverative errors. Instead, both anticipatory and perseverative errors decreased. The psychometric function was step-like, suggesting that pigeons were primarily relying on response-outcome cues. Moreover, studies that varied the reversal trial randomly across sessions—making timing less reliable—provided evidence of joint control by timing and reinforcement cues in pigeons (Rayburn-Reeves et al., 2011; Santos et al., 2021) and rats (Santos and Sanabria, 2020; Smith et al., 2017).

If it is clear that different cues influence the pigeons’ choices in the MSR task, how they are integrated or combined remains to be investigated (see McMillan, Spetch, et al., 2017; Rayburn-Reeves & Cook, 2016; Santos et al., 2021; Zentall, 2020). In an effort to understand how the different sources combine, two dynamic models of learning have been recently proposed (Santos et al., 2019, 2021; Santos & Sanabria, 2020). Both models assume that, on each trial, animals enter either a timing mode, with probability *p*, or a nontiming mode with probability 1-*p*. How the timing mode is conceived varies between the models: In the first (i.e., Mixture Model I, Santos et al., 2021), the timing mode instantiates a generic pacemaker-accumulator unit as in scalar expectancy theory (e.g., Gibbon et al., 1984; Meck & Church, 1983), whereas, in the second (i.e., Mixture Model II, Santos et al., 2021), the timing mode follows the learning-to-time model (LeT; Machado, 1997; Machado et al., 2009). The nontiming mode is similar in both models and follows a win-stay/lose-sometimes-shift strategy according to which a response will be repeated until it has been unreinforced for a particular number of consecutive trials. Santos et al. (2021) ran simulations with both models and found that the second produced results closer to those observed with pigeons. Even though the model integrates two of the sources of control mentioned above, the global cue of time into the session and the local cue of response outcomes (extinction in particular) of the last trials, it does not include control by number of trials. Our results have implications for theory and experiment. Improved models of the MSR task must include trial number as a source of behavioral control, together with time into the session and local response-outcome events. Further experiments should unravel how and when each of these cues influence behavior (e.g., cue competition, hierarchical organization of discriminative cues).

Another point of interest for future research is identifying which temporal (and counting) markers pigeons are using as well as the limits of each strategy. As an illustration, in an exploratory unpublished study on the limits of the timing strategy, we trained pigeons P916 and P890 in a MSR task with a longer ITI (60 s; session duration ≈ 1 h 20). With significantly longer ITIs, time- and number-based strategies should become more difficult making win-stay/lose-shift strategy more attractive. The psychometric functions of both pigeons showed anticipatory and perseverative errors, a pattern consistent with the use of a global cue. In the last session of training, for P916 the trial-based PSE equaled 46 and it occurred 46 min into the session; for P890, the trial-based PSE equaled 40 and it occurred 40 min into the session. Then, in a test session with all responses reinforced, the ITI was halved (30 s). Results suggested the use of a timing cue: P916 reversed on trial 84, 43 min into the session, and P890 reversed on trial 69, 38 min into the session, both reversals happening approximately at the same time as they did in training. These preliminary data show that the reliance on a timing strategy is quite robust, and further studies are needed to clarify its boundary conditions.

In summary, results from different studies and with different species have shown that there are different sources of behavioral control in the MSR task and that animals might alternate between cues or combine them to respond. In this study, we tested the timing hypothesis by changing the ITI duration from training to testing and removing the extinction cue that seemed problematic in previous studies (e.g., McMillan & Roberts, 2012; Soares et al., 2020). In Condition I, we reinforced all choices during the test session, but that introduced a different nuisance factor, the reinforcement of early S2 choices, which may have promoted early switching and indifference between the two options. This result in itself evinces that pigeons, even when following a timing strategy remain sensitive to local reinforcement contingencies. To eliminate this nuisance factor from the test session, we modified the reinforcement rules so that trial number or time-into-the-session could exert control and not be preempted by contingency cues: When the ITI increased, S2 responses were reinforced only after trial 20 and, when the ITI decreased, S2 responses were reinforced only after trial 40. Under these conditions, we found that (most) pigeons’ behavior accorded with a timing account: In both conditions, the psychometric functions shifted in the expected direction and close to the expected magnitude. The fact that the shifts were slightly smaller than expected suggests there may still be other effects at play, such as generalization decrement effects.

With procedural refinements, the present study clarified the cause of the discrepancy previously found between tests with halved and doubled ITIs. Conditions I and II of the present study (as well as previous experiments) are a good demonstration of the difficulty of testing temporal control without introducing additional cues. The same difficulties occur when testing for stimulus generalization after discriminative training: Should the responses to the new, untrained stimuli be extinguished or nondifferentially reinforced? If the latter, should the reinforcement rate be the same or lower than the rate during training? Perhaps the best way to address these questions is to examine the results obtained under various testing procedures, attempt to integrate and interpret them, and assess if convergent evidence arises. We followed this approach in the present study: By testing the timing hypothesis in two conditions, we clarified the test-asymmetry effect and strengthened the evidence in its favor, while also raising new questions that must be addressed, namely the finding that some pigeons solved the task seemingly by counting, a result that challenges all current models of the MSR task.

## Appendix

**Individual psychometric functions**
